# Biochemical analysis of GTPase FlhF which controls the number and position of flagellar formation in marine *Vibrio*

**DOI:** 10.1038/s41598-018-30531-5

**Published:** 2018-08-14

**Authors:** Shota Kondo, Yoshino Imura, Akira Mizuno, Michio Homma, Seiji Kojima

**Affiliations:** Division of Biological Science, Graduate School of Science, Nagoya University, Chikusa-ku, Nagoya, 464-8602 Japan

## Abstract

FlhF controls the number and position of the polar flagellar formation of *Vibrio* species. FlhF, is a paralog of FtsY, a GTPase acting in the Sec membrane transport system of bacteria, and localizes at the cell pole. Mutations in the conserved GTPase motif of FlhF lost polar localization capability and flagellar formation. *Vibrio* FlhF has not, until now, been purified as soluble protein. Here, we report that addition of MgCl_2_ and GTP or GDP at the step of cell lysis greatly improved the solubility of FlhF, allowing us to purify it in homogeneity. Purified FlhF showed GTPase activity only in the presence of FlhG. Of twelve FlhF GTPase motif mutants showing reduced function, eleven were recovered as precipitate after the cell disruption. The E440K substitution could be purified and showed no GTPase activity even in the presence of FlhG. Interestingly an FlhF substitution in the putative catalytic residue for GTP hydrolysis, R334A, allowed normal flagellar formation although GTPase activity of FlhF was completely abolished. Furthermore, size exclusion chromatography of purified FlhF revealed that it forms dimers in the presence of GTP but exists as monomer in the presence of GDP. We speculate that the GTP binding allows FlhF to dimerize and localize at the pole where it initiates flagellar formation, and the GDP-bound form diffuses as monomer.

## Introduction

Many motile bacteria have filamentous protrusion organelles called flagella on the cell surface. It allows cells to swim in liquid by rotating their flagella like a screw. Among the bacterial species, the number and position of flagellar formation varies. For example, *Escherichia coli* and *Bacillus subtilis* have multiple, peritrichous flagella around the cell body while *Campylobacter* has a single flagellum at both poles, and *Pseudomonas aeruginosa* and *Vibrio* species have a single flagellum at one pole. FlhF and FlhG are proteins that control flagellar formation^1–3^. Overexpression of FlhF in a marine bacterium, *Vibrio alginolyticus*, which has a single polar flagellum, results in the synthesis of multiple polar flagella while the loss of FlhF results in the absence of a flagellum. Thus, FlhF positively controls flagellar number^3^. On the other hand, when FlhG is overexpressed, cells lack a flagellum whereas multiple flagella are produced at cell pole when FlhG is absent. Thus, FlhG negatively controls the number of flagella formed. FlhF is important for determining the position of flagellar formation at the cell pole. The polar localized FlhF promotes flagellar formation, and FlhG interferes with the polar localization of FlhF^4,5^. FlhF is localized at cell pole by itself, but FlhG localizes to the pole via the scaffolding membrane protein HubP and seems to inhibit the FlhF function directly^6^. The detailed mechanisms of flagellar formation controlled by FlhF and FlhG have not been clarified.

FlhG is a protein with an ATPase motif homologous to MinD, which is known as a factor controlling cell division in *Escherichia coli*. MinD constitutes the Min system together with MinC and MinE to control FtsZ ring formation at the cell division plane^7^. We made ATPase motif mutants in *V. alginolyticus* FlhG and found that the ATPase motif of FlhG plays an important role in the inhibition of FlhF function^8^. Furthermore, we have purified FlhG and measured its ATPase activity, showing that ATP-dependent polar localization of FlhG is crucial for its ability to regulate the number of polar flagella. We speculate that ATP hydrolysis by FlhG is required for the fine-tuning of its ability to regulate flagellar number at the pole. On the other hand, FlhF shows homology to FtsY and Ffh, which are GTPase that act to target membrane proteins of *E. coli* to the cytoplasmic membrane. Those membrane proteins have a hydrophobic Sec-dependent signal sequence to direct their insertion into the cytoplasmic membrane. Soon after the signal sequence is exposed from the ribosome during nascent protein synthesis, Ffh in the signal recognition particle (SRP) binds to the signal sequence. Then the SRP-nascent protein complex is bound to the SRP receptor FtsY and targeted to the Sec machinery^9,10^. FtsY and Ffh interact with each other by binding GTP to form an Ffh/FtsY complex and the ongoing polypeptide in the ribosome is guided to the Sec transport machinery. Both FtsY and Ffh have a GTPase motif, and the Ffh/FtsY complex dissociates by hydrolyzing GTP to become the GDP-bound form. The binding of FtsY and Ffh to GTP and the GTPase activity play an important role in a series of processes for targeting membrane proteins to membranes^11,12^.

FlhF has the conserved GTPase motifs I to IV which play an important role in GTP hydrolysis^13^. In *Bacillus subtilis*, the crystal structure of FlhF has been solved in a form of GTP-bound homodimer^14^. The GTPase activity of *B. subtilis* FlhF was detected and stimulated by FlhG^11^. In *Campylobacter jejuni, Pseudomonas aeruginosa*, *Shewanella oneidensis* and *Shewanella putrefaciens*, the GTPase activity was detected in FlhF^15–18^. In *C. jejuni*, the GTPase motif mutants that decrease the GTPase activity altered the flagellar number and the position of flagellar formation on the cell body^15^. On the other hand, in *Vibrio cholerae*, the mutation of the putative catalytic residue for GTP hydrolysis did not affect flagellar formation, whereas mutations that abolished GTP-binding ability did affect flagellar formation^19^. This suggests that GTP binding is essential for FlhF function but GTPase activity is dispensable. The phenotypes of the GTPase motif mutants might be different among the bacterial species.

In an earlier study^20^, we investigated eight *V. alginolyticus* FlhF mutants with amino acid substitutions in motifs I, III and IV (G299A, G304A, K305A, T306A, D377A, G380A, K437A, D439A, please see also Fig. S1). The T306A and D439A substitutions completely lost the ability to synthesize flagella. The G299A, G304A, K305A, D377A, and K437A mutants could form flagella but with a reduction in both flagellar number and polar localization of FlhF. The G380A mutant did not substantially decrease flagellar formation and was more strongly localized to the cell poles, but its motility was reduced. We also isolated *flhF* mutants by random mutagenesis to select for those with a polar localization deficient phenotype^21^. As a result, two point mutants (T436M and E440K) were isolated, both of which are located in GTPase motif IV. The T436M and E440K mutants completely lost the ability to form flagella, and their polar localization was greatly reduced as compared to the wild type. This strongly suggested that the GTPase motif was important for the polar localization of FlhF and the initiation of polar flagellation. Phenotypic analysis of *V. alginolyticus* FlhF has been extensively performed, however its biochemical characterization has been hampered because overproduced FlhF in *E. coli* cells precipitated after cell lysis. In this study, we tested various conditions to obtain soluble FlhF protein and establish a method for its purification, which would allow for the characterization of its biochemical properties. Based on the results obtained in this study, we present a model of the mechanism of flagellar regulation by FlhF.

## Results

### Purification of FlhF

**Figure 1:**
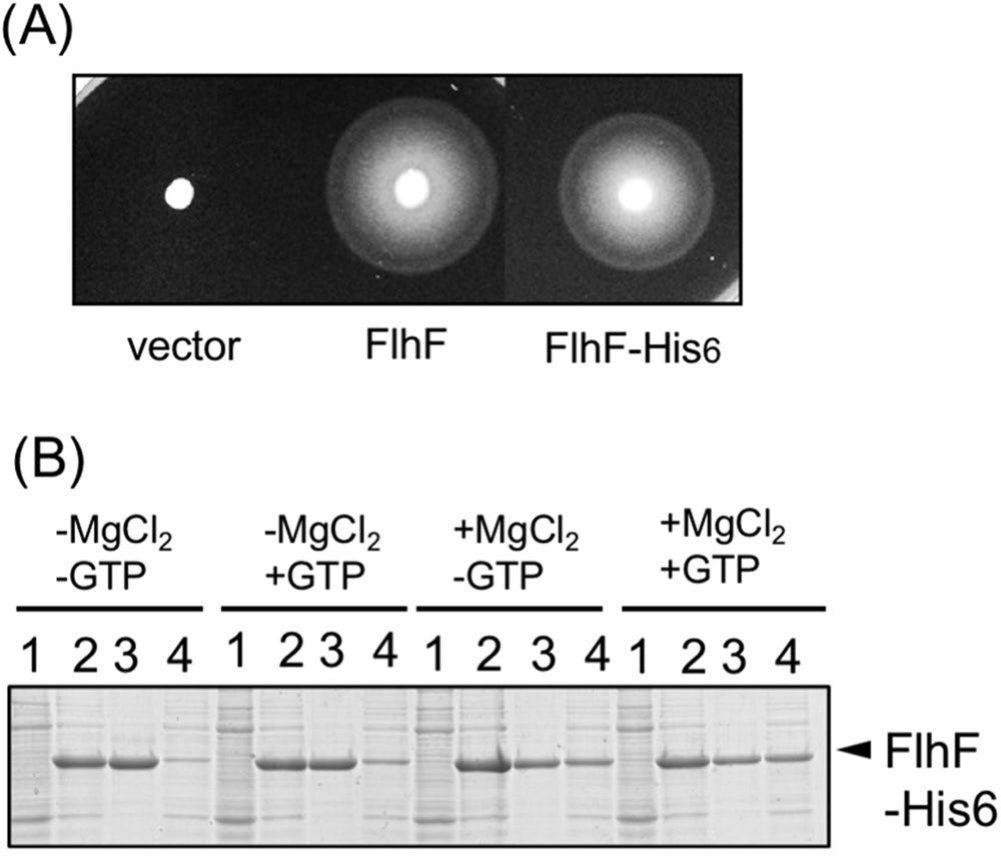
Characterization of FlhF protein. (**A**) Cell motility. Cells of the *flhF* mutant (LPN1) harboring a plasmid, vector; pBAD33, FlhF; pAK322, or FlhF-His6; pTSK119, were inoculated into a soft agar plate containing 0.02% arabinose and incubated for 6 hours at 30 °C. (**B**) Cells of BL21(DE3) harboring a plasmid pTSK110 which has *flhF-his6* in pColdIV vector were broken in the presence and absence of MgCl_2_ or GTP. The proteins were separated by SDS-PAGE and the gels were stained by CBB. The regions of interest were cropped from the gels. 1: the pre-induction cells, 2: the post-induction cells, 3: the low speed centrifugal precipitation, 4: the low speed supernatant.

**Figure 2:**
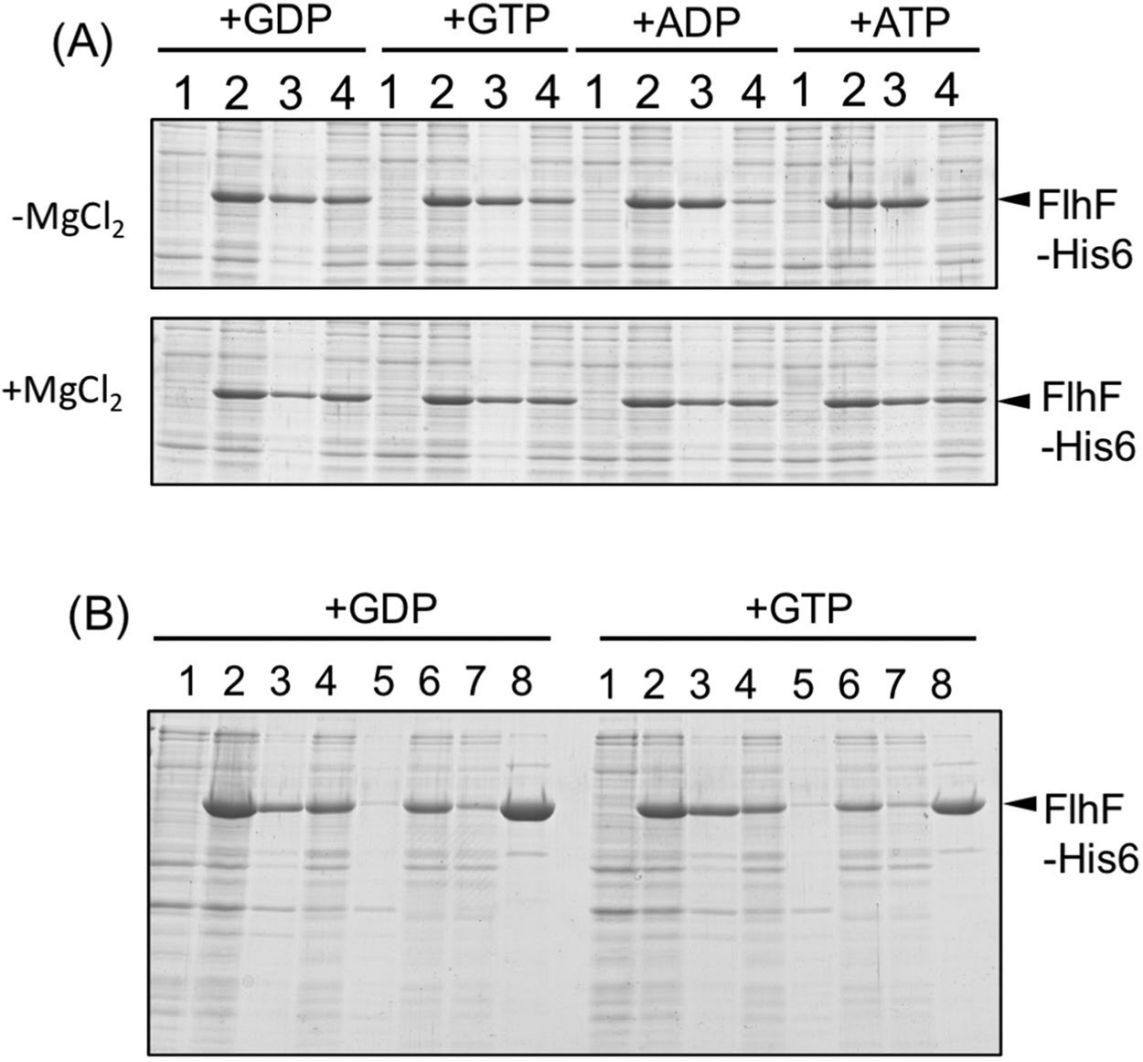
Purification of FlhF. (**A**) Cells of BL21(DE3) harboring a plasmid pTSK110 which has *flhF-his6* in pCold IV vector were broken in the presence of GDP, GTP, ADP and ATP with (lower panel) or without (upper panel) MgCl_2_. The proteins were separated by SDS-PAGE and the gels were stained by CBB. The regions of interest were cropped from the gels. 1: the pre-induction cells, 2: the post-induction cells, 3: the low speed centrifugal precipitation, 4: the low speed supernatant. (**B**) Purification of FlhF by adding GTP or GDP. 1: the pre-induction cells, 2: the post-induction cells, 3: the low speed centrifugal precipitation, 4: the low speed supernatant, 5: the ultracentrifugation precipitation, 6: the post-ultracentrifugation supernatant, 7: the flow through fraction of Ni-NTA resin, 8: the eluted fraction of Ni-NTA resin. The proteins were separated by SDS-PAGE and the gels were stained by Coomassie brilliant blue (CBB).

Purification of *V. alginolyticus* FlhF had been carried out using *E. coli* BL21 (DE3) transformed by the plasmid pET-FlhF, which overexpresses His6-FlhF from T7 promoter^4^. In that study, FlhF inclusion bodies were pelleted by low speed centrifugation followed by denaturation by treatment with urea and then affinity purified. However, when urea was removed FlhF again precipitated. In this study, we attached His6 tag to the C-terminus of *V. alginolyticus* FlhF resulting in a protein with a total length of 511 amino acids and a molecular weight of 57.4 kDa (Fig. S1). Plasmid expressed FlhF-His6 or FlhF showed similar motility in the *flhF* mutant strain (LPN1) strain (Fig. 1A), and flagellation was observed in both strains by a high-intensity dark-field microscope. This demonstrated that FlhF-His6 was functional in *Vibrio* cells. His-tagged FlhF was then produced in *E. coli* using a cold shock expression vector pCold IV, which allowed overexpression from the *cspA* promoter. Although we anticipated that low-temperature expression might improve protein stability, almost all of the FlhF protein precipitated out following low speed centrifugation after cells were broken by sonication in T-buffer [50 mM Tris-HCl (pH 8.0), 150 mM NaCl] (Fig. S2A). However, a small amount of FlhF protein remained in the supernatant after low-speed centrifugation that was capable of affinity purification. We investigated the conditions that FlhF could be recovered in the supernatant following low speed centrifugation. When 1 mM GTP was added to the T-buffer before sonication, more FlhF was recovered in the supernatant following low speed centrifugation (Fig. 1B, −MgCl_2_/+GTP). The intensity of the FlhF bands after the low speed centrifugation was measured, and its ratio (supernatant/pellet) increased in the presence of GTP (0.2 for −MgCl_2_/−GTP, but 0.3 for −MgCl_2_/+GTP). Therefore the stability of FlhF protein seemed to increase in the presence of GTP. We also tested the effect of Mg^2+^ since FtsY of *E. coli* as well as FlhF of *Bacillus subtilis* and *Campylobacter* were successfully purified by adding Mg^2+^ to buffer^4,15,22^. Indeed, addition of Mg^2+^ increased the recovery yield of His-tagged FlhF (Fig. 1B, +MgCl_2_/−GTP, sup/ppt ratio is 0.64). About half of the FlhF protein produced was recovered in the supernatant fraction when both GTP and MgCl_2_ were added to the T-buffer before sonication (Fig. 1B, +MgCl_2_/+GTP, sup/ppt ratio is 0.99). Therefore, cells were sonicated in a buffer containing 0.5 mM GTP and 10 mM MgCl_2_, and purification was performed in the presence of 10 mM MgCl_2_ (Fig. S2B). The purified His6-FlhF (hereafter, simply FlhF) protein aggregated when left at room temperature or at 4 °C for several hours. However, the aggregation was not observed if 10% (w/v) glycerol was added into the elution buffer. Since the addition of GTP to the buffer improved the purification of FlhF, we examined the effects of adding other nucleotides: GDP, ATP and ADP. We found that GDP was more effective for recovering soluble FlhF protein (Fig. 2A). Therefore, purification was carried out using buffer containing 0.5 mM GDP and 10 mM MgCl_2_ (Fig. 2B).

A scale-up of FlhF purification was attempted from 100 ml of cell culture by the small-scale procedure described above. FlhF was recovered in the supernatant fraction following low-speed centrifugation and ultracentrifugation. However, when the ultracentrifuged supernatant was added to Ni-NTA resin and mixed by a rotator, the large amount of white precipitate was formed and FlhF was lost. Thus, to shorten the processing time, immediately after ultracentrifugation, the supernatant was applied to a disposable column packed with Ni-NTA resin, followed by immediate washing and elution with imidazole. As the result, almost all the FlhF protein adsorbed to the Ni-NTA resin without precipitation and was successfully eluted from the column (Fig. S3). From 100 ml of culture, 2.5 ml of purified FlhF protein solution was obtained at ca. 0.5 mg ml^−1^.

### Measurement of the GTPase activity of FlhF

**Figure 3:**
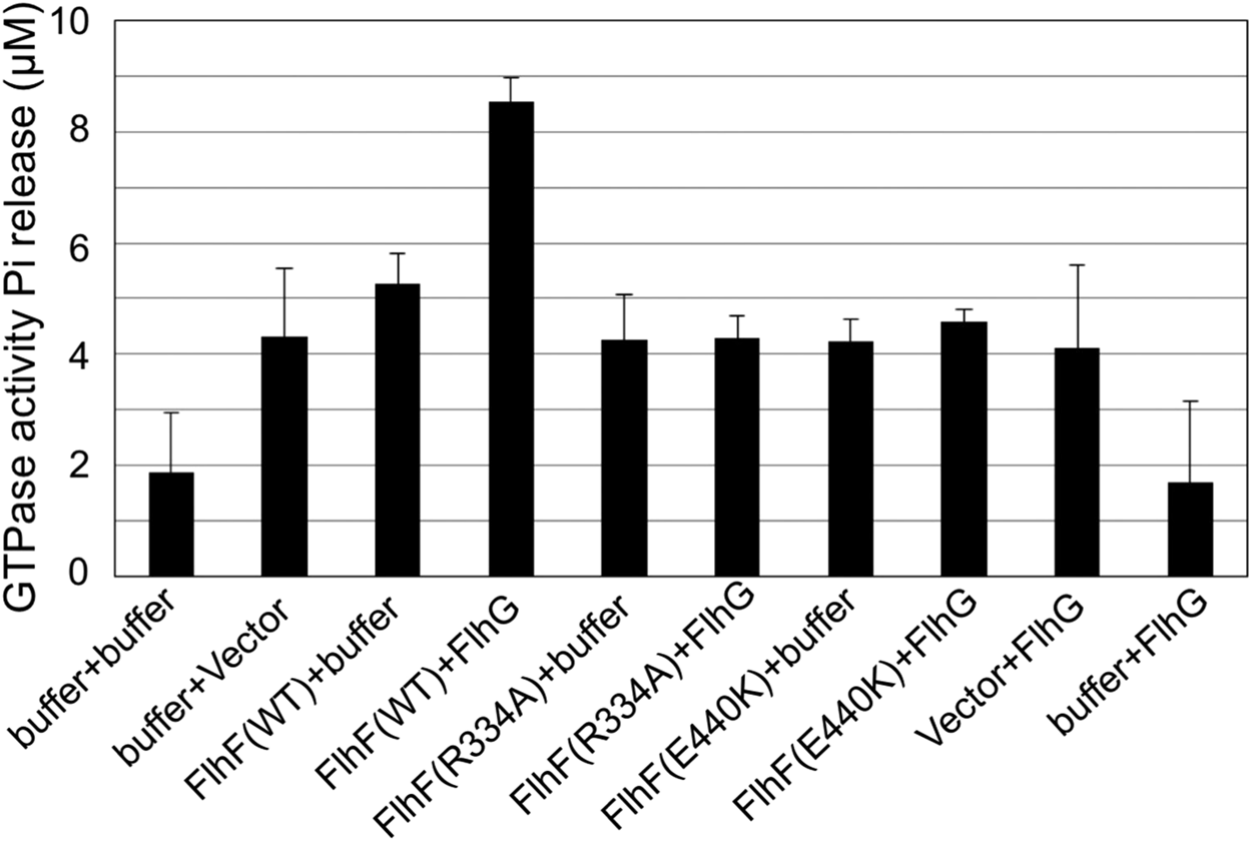
The GTPase activity of purified FlhF. The amount of inorganic phosphate release was measured in a mixture of 1.0 μM FlhF, 0.5 mM GTP, and 10 mM MgCl_2_ for 30 min. To examine the effect of FlhG on FlhF-GTPase, 1.0 μM FlhG was also added in the reaction. The sample solutions were prepared from BL21(DE3) cells harboring a plasmid pCold IV; Vector, pTSK110; FlhF(WT), pSK313; FlhF(R334A), or pSK311; FlhF(E440K), and of BL21(DE3)/pLysS harboring a plasmid pTrc-flhG; FlhG. The activity was measured by mixing of FlhG buffer containing FlhF and GTP buffer containing FlhG for three times with different purification batches. Error bars are standard deviation.

GTPase activity was measured using the purified FlhF protein in order to determine the amount of inorganic phosphate released by GTP hydrolysis. Because purified FlhF was slightly contaminated with other proteins, the background GTPase activity was measured using the sample that was prepared by the same purification procedure from cells containing only vector plasmid. 1 μM FlhF released 5.3 μM inorganic phosphate in 30 minutes, while the background sample released 4.3 μM inorganic phosphate (Fig. 3). This indicated the presence of significant background GTPase activity in addition to that from FlhF. However, when purified FlhF was mixed with purified FlhG at the same concentration, the released inorganic phosphate increased significantly to 8.5 μM (Fig. 3). When FlhG was added in the background sample or buffer, the amount of inorganic phosphate release did not increase. This shows that FlhG is necessary for stimulation of the FlhF GTPase activity.

### Purification of GTPase motif mutants

**Figure 4:**
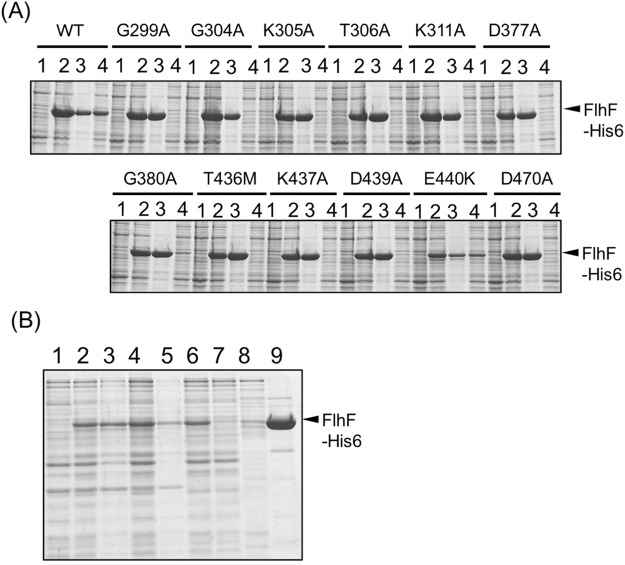
Characterization and purification of the mutant FlhF proteins. (**A**) Cells of BL21(DE3) harboring a plasmid pTSK110(WT) or the derivative mutant plasmids carrying *flhF-his6* in pCold IV vector were broken in the presence of GDP. The proteins were separated by SDS-PAGE and the gels were stained by CBB. The regions of interest were cropped from the gels. 1: the pre-induction cells, 2: the post-induction cells, 3: the low speed centrifugal precipitation, 4: the low speed supernatant. (**B**) BL21(DE3) cells harboring a pTSK110 derivative mutant plasmid (E440K) were broken in the presence of GDP. 1: the pre-induction cells, 2: the post-induction cells, 3: the low speed centrifugal precipitation, 4: the low speed supernatant, 5: the ultracentrifugation precipitation, 6: the post-ultracentrifugation supernatant, 7: the flow through fraction of Ni-NTA resin, 8: the washed fraction of Ni-NTA resin, 9: the eluted fraction of Ni-NTA resin. The proteins were separated by SDS-PAGE and the gels were stained by CBB.

Our previous studies showed that GTPase motif mutants in FlhF resulted in defects in FlhF polar localization and flagellar formation^20,21^. Twelve mutants (G299A, G304 A, K305A, T306, K311A, D377A, G380A, T436M, K437A, D439A, E440K, and D470A) were examined. In an attempt to purify the twelve mutant proteins as described above for wild-type FlhF, we found that all but E440K precipitated out following the low speed centrifugation step (Fig. 4A). Although the expression level of E440K was lower than that of the wild type, we succeeded in purifying it to a concentration of ca. 0.3 mg ml^−1^ (Fig. 4B).

The E440K mutant was obtained by a random mutagenesis^21^. We confirmed that the E440K mutant could not complement the motility defect of the LPN1 *flhF* null mutant cells. Motility was completely absent on soft agar medium and flagellar formation was not observed by high-intensity dark field microscope. The E440K mutant FlhF fused with GFP was expressed in the *flhF* null mutant background and observed by fluorescence microscopy. The polar localization ratio of wild-type FlhF-GFP was ~87%, whereas the localization ratio of FlhF (E440K)-GFP was ~33%. The GTPase activity of E440K mutant was the same as that from background vector which is only cell extracts (4.2 μM inorganic phosphate in 30 minutes, Fig. 3). This indicated that the E440K mutant lost GTPase activity.

### Purification of FlhF mutant of putative GTPase catalytic residues

**Figure 5:**
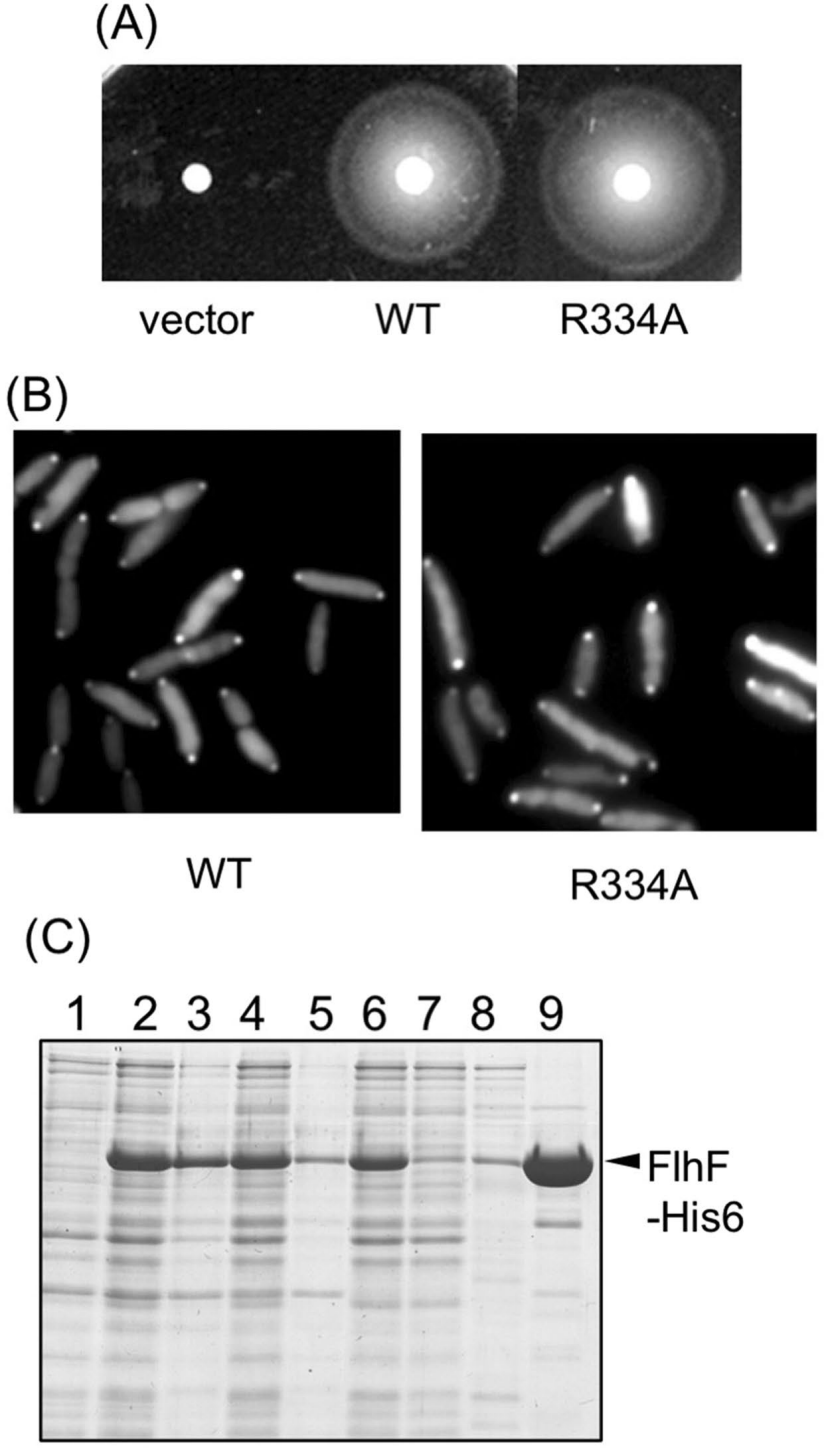
GTPase activity is dispensable for the FlhF function. (**A**) Cells of the *flhF* mutant (LPN1) harboring a plasmid, vector; pBAD33, WT; pAK322, or R334A; pSK111, were inoculated into a soft agar plate containing 0.02% arabinose and incubated for 6 hours at 30 °C. (**B**) Cells of the *flhF* mutant (LPN1) harboring a plasmid, WT; pAK325, or R334A; pSK211, were inoculated in VPG medium containing 0.02% arabinose and observed with a fluorescence microscope. (**C**) BL21(DE3) cells harboring a pTSK110 derivative mutant plasmid (R334) were broken in the presence of GDP. 1: the pre-induction cells, 2: the post-induction cells, 3: the low speed centrifugal precipitation, 4: the low speed supernatant, 5: the ultracentrifugation precipitation, 6: the post-ultracentrifugation supernatant, 7: the flow through fraction of Ni-NTA resin, 8: the washed fraction of Ni-NTA resin, 9: the eluted fraction of Ni-NTA resin. The proteins were separated by SDS-PAGE and the gels were stained by CBB.

To further investigate the role of FlhF GTPase activity on its polar localization and flagellar formation, an R334A substitution was constructed in the putative catalytic residue for GTP hydrolysis. The corresponding residue of *P. aeruginosa* FlhF is R251 and its GTPase activity was lost by an R251G substitution^16^. When the R334A mutant was produced from our plasmid expression system in the *flhF* mutant strain LPN1, motility was comparable to wild type FlhF on soft agar medium (Fig. 5A). Its flagellar formation was also similar to that of wild type FlhF as observed by high-intensity dark field microscope. When R334A FlhF-GFP was expressed in the *flhF* mutant strain, fluorescent dots were observed at the cell pole in ca. 84% cells as was observed with wild-type FlhF-GFP (Fig. 5B). Overproduced R334A FlhF protein could be purified similar to the wild-type protein (Fig. 5C). The concentration of R334A FlhF protein was 0.42 mg ml^−1^. We used this protein to measure the GTPase activity and the inorganic phosphate was released at the background level even in the presence of FlhG (Fig. 3). The mutant R334A gave the similar polar localization phenotype as wild-type FlhF, but the GTPase activity of R334A was lost, suggesting that the GTPase activity is not necessary for the FlhF function.

### Size estimation by gel filtration chromatography

**Figure 6:**
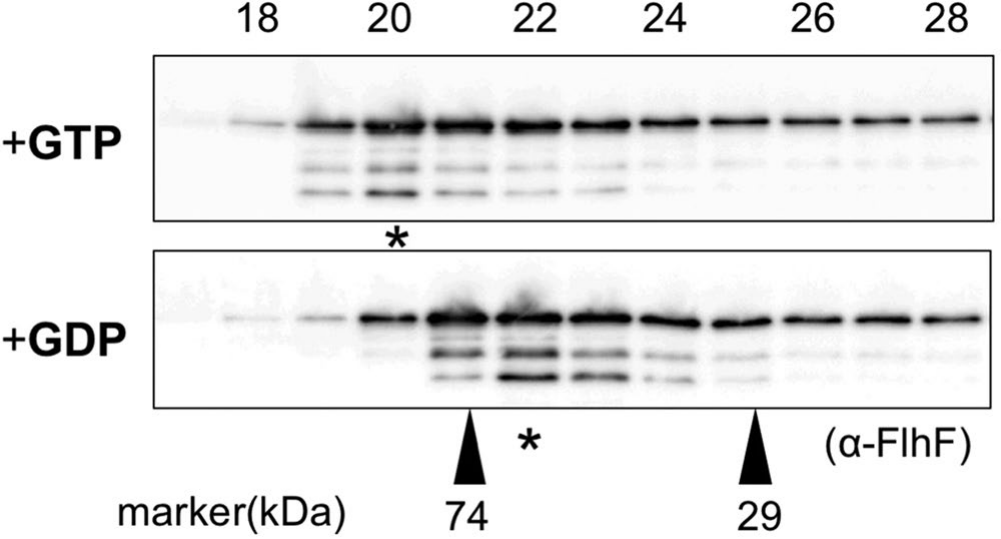
FlhF forms a dimer in the presence of GTP. FlhF was incubated in the presence of either 1 mM GTP or GDP and analyzed by gel filtration chromatography using buffer containing either 1 mM GTP or GDP. The upper panel shows the profile in the presence of GTP, and the lower panel shows it in the presence of GDP. Immunoblotting was performed for each fraction using the anti-FlhF antibody. The regions of interest were cropped from the immunoblotting. The peak fraction containing FlhF is indicated by the asterisk. The molecular size markers were carbonic anhydrase (29 kDa) and conalbumin (74 kDa).

It is known that FtsY and Ffh form a heterodimer upon binding GTP and the crystal structure of *B. subtilis* FlhF GTP-bound homodimer has been solved. Thus, we speculated that *V. alginolyticus* FlhF also forms a GTP-bound homodimer. To examine this, we performed gel filtration chromatography to estimate size changes of FlhF in the presence of either GTP or GDP (Fig. 6). FlhF eluted in fraction 20 in the presence of GTP, which corresponded to the size of the FlhF dimer (ca. 114 kDa). On the other hand, in the presence of GDP, FlhF eluted in fraction 22, which corresponded to the size of an FlhF monomer (ca. 57 kDa). These results demonstrated that *V. alginolyticus* FlhF forms a dimer in the presence of GTP and exists as a monomer in the presence of GDP.

## Discussion

*V. alginolyticus* FlhF is a soluble protein, and is required to initiate flagellar formation and to regulate flagellar number in the presence of FlhG. FlhF and FlhG belong to the SIMIBI family (the nucleotide triphosphate binding protein family essential for protein targeting in a wide range of cellular processes). The SIMIBI proteins act as molecular switches with the NTP binding “on” state and the NDP binding “off” state^23^. *V. alginolyticus* FlhF aggregates easily, precipitating after low speed centrifugation following cell disruption. Thus, purification of *Vibrio* FlhF has proved challenging. In this study, FlhF was produced at low temperature using a cold shock expression vector and cells were disrupted in a buffer containing MgCl_2_ and GTP or GDP. by this method, soluble FlhF was recovered following low speed centrifugation. This may be due to stabilization of FlhF in the presence of Mg^2+^ or nucleotides. In *Bacillus subtilis*, the crystal structure of FlhF was solved bound with Mg^2+^ and nucleotides^14^. Therefore, we presume that *V. alginolyticus* FlhF binds to GTP or GDP with Mg^2+^ and the nucleotide binding state is stable. The GTP binding motif mutants, except for E440K, precipitated following low speed centrifugation. Mutations of the GTP binding motif probably affects the structure around the motif.

SIMIBI proteins form dimers in the NTP-bound form and play regulatory roles in various cellular processes such as cell division and protein transport^23^. FtsY and Ffh and MinD of *E. coli* belong to this family and form heterodimers in either a GTP-dependent manner for FtsY and Ffh or an ATP-dependent manner for MinD. Their crystal structures have been solved. In this study, analysis by gel filtration chromatography suggested that *V. alginolyticus* FlhF also forms a dimer in the presence of GTP and is stably present as a monomer in the presence of GDP. This may explain why more soluble FlhF could be recovered following low-speed centrifugation in disruption buffer containing GDP than buffer containing GTP. Higher ordered multimer formation is more likely to promote aggregation. We think that dimer formation is important for polar localization.

Until this study, *V. alginolyticus* FlhF had not been purified in its native form. We could detect GTPase activity, however, FlhF by itself only showed GTPase activity in the presence of FlhG. The putative catalytic residue mutant R334A lacked GTPase activity in the presence of FlhG though its intracellular localization and the formation of flagella were similar to wild type. These results suggest that the GTPase activity of FlhF is not necessary for the polar localization and the formation of flagella. Since the GTP-binding motif mutant E440K lost its GTPase activity and conferred an abnormal subcellular localization of FlhF and loss the ability to form flagella, the GTP binding seems to be important for polar localization of FlhF and flagellar formation. However, it has not been confirmed whether the purified wild-type FlhF actually binds GTP and whether the R334A mutant can bind GTP but the E440K mutant cannot.

It has been speculated that FlhF interacts to recruit the FliF protein to the cytoplasmic membrane to initiate the construction of the flagellar MS-ring structure, which is composed of FliF^19^. We have shown that FlhF localizes at the cell pole of *rpoN*-deficient strain of *V. alginolyticus* as well as in the *flhDC*-deficient strain of *E. coli*^4^. Therefore, we can conclude that FlhF by itself has an ability to localize at the cell pole, though FlhG needs HubP, which is a single transmembrane protein with an N-terminal periplasmic peptidoglycan-binding motif (LysM) and a large cytoplasmic domain^24^, to localize at the cell pole^6^. The GDP-bound form of FlhF diffuses around the cytoplasm as monomer and forms a dimer by binding with GTP to acquire the ability to localize the pole. How bacteria localize proteins to cell pole is not clear^25–27^. For example, chemoreceptors involved in chemotaxis of *E. coli* are located at cell poles^28^ and it has been recently shown that they become stably present at highly curved cell poles by forming multimers^29^. It has been shown that *Caulobacter crescentus* uses a multimeric pole-organizing factor (PopZ) that serves as a hub to concurrently achieve several polarizing functions and the polar accumulation of PopZ is central to its polarizing activity^30^. We speculate that a mechanism similar to that used by PopZ is employed by FlhF to localize to cell poles. Perhaps FlhF localizes at cell poles by forming a dimer, and the dimer might form multimers to retain polar localization. To initiate the flagellar assembly, it is inferred that FlhF assists in MS-ring formation at the poles.

It is noteworthy that FlhF is not essential for flagellation: very few cells of Δ*flhF* strain can still form a flagellum, and the *flhF* and *flhG* double mutant slightly increases the number of flagellated cells. When the additional defect of the membrane factor, SflA, is present in the Δ*flhFG* background, more than 50% of cells can generate multiple flagella at lateral positions^31^. The initiation of flagellar formation seems to be suppressed by SflA in the absence of FlhF. SflA is localized at cell poles, dependent on HubP^32^. HubP does not belong to the flagellar regulon and may be regulated independent from the flagellar genes. It was suggested that HubP is involved in the regulation of chromosome partitioning and chemoreceptor positioning in *V. cholerae*. It has been shown in *V. cholerae* and *P. aeruginosa* that FlhG (FleN) also functions as a transcriptional repressor of the polar flagellar genes^2,33^ and protein production of the polar flagellar genes increased in *flhG* deficient strain of *V. alginolytius*^4^. In FlhG (FleN) of *P. aeruginosa*, the reduction in activity of the master transcriptional regulator is dependent on ATP and its ATPase domain^33,34^.

**Figure 7:**
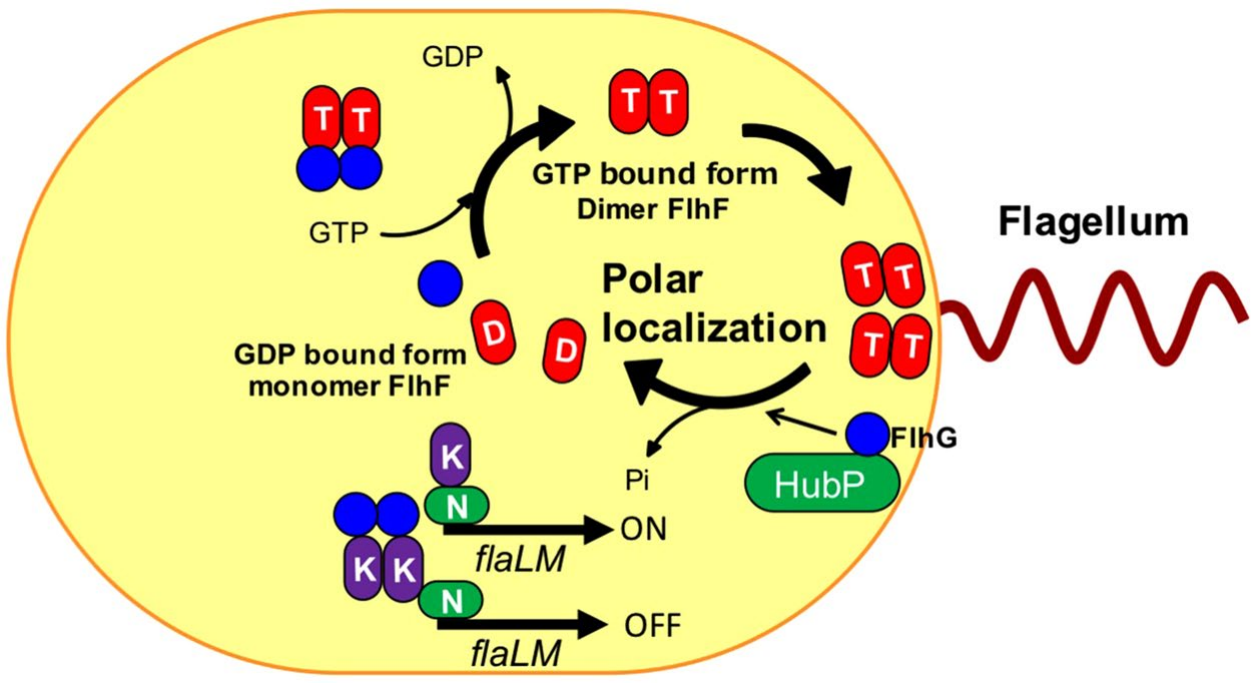
The model of FlhF polar localization in *V. alginolyticus*. FlhF diffuses into the cytoplasm as a GDP-bound monomer (shown as the red ellipse with “D”), but when it binds to GTP (shown as the red ellipse with “T”), it forms a dimer and becomes localized to the pole. Localized FlhF promotes flagellar formation at cell pole. FlhG diffuses in the cytoplasm and interacts with FlhF to inhibit polar localization of FlhF. Also, FlhG binds to FlaK (shown as the purple ellipse with “K”) to inhibit the early class of flagellar genes (*flaLM*), whose expression is dependent on FlaK and RpoN (shown as the green ellipse with “N”). At the poles, the GTPase activity of FlhF is activated by FlhG, which is localized to the pole by the HubP scaffold. FlhF is converted to the GDP bound form and dissociates from the cell pole. In the wild type *V. alginolyticus*, the amount of FlhF localized at the pole is controlled to a level resulting in biosynthesis of a single, polar flagellum.

Based on the above evidence and previous reports, we present a model on how FlhF controls flagellar formation (Fig. 7). FlhG appears to negatively control flagellation by activation of FlhF GTPase activity. Since FlhG can exist throughout the cytoplasm and at the cell pole, it is possible that FlhG activates FlhF GTPase activity at both places to produce a GDP-bound or unbound form of FlhF that can no longer stay at the pole and is retained throughout the cytoplasm. As mentioned above, GTP hydrolysis activated by FlhG at the pole, but is not necessary to induce the release of FlhF from the pole. The GTP-bound form of FlhF by itself might be able to associate to the polar region and dissociate from the pole by the effects of FlhG whose polar localization is dependent on HubP. This is consistent with the fact that overexpression of FlhG in cells did not reduce the polar localization rate of FlhF and the *hubP* mutant gave the multi polar phenotype similar to the phenotype of the *flhG* mutant^6^. As suggested above, FlhF is likely to recruit the FliF to the pole and may also act on the formation of the FliF MS-ring. To unravel the mechanism of initiation for polar flagellar assembly, it will be necessary to investigate the dynamic interactions of proteins at the pole including FlhF, FlhG, FliF and HubP. For this purpose, it would be helpful to solve the crystal structure of *V. alginolyticus* FlhF though it has been determined in the homologous proteins, such as FlhF homolog of *B. subtilis*^14^ and FtsY or Ffh paralog of *E. coli*^35^.

### Experimental procedures

#### Strains used, culture medium and culture conditions

The strains and the plasmids used in this study are shown in Table 1. *E. coli* cells were cultured at 37 °C in LB medium [1% (w/v) bactotryptone, 0.5% (w/v) yeast extract, and 0.5% (w/v) NaCl]. The gene under the control of the cold shock gene *cspA* promoter of *E. coli* was induced by the addition of IPTG at low temperature (16 °C). *V. alginolyticus* cells were cultured at 30 °C in the VC medium [0.5% (w/v) polypeptone, 0.5% (w/v) yeast extract, 0.4% (w/v) K_2_HPO_4_, 3% (w/v) NaCl, and 0.2% (w/v) glucose] or VPG medium [1% (w/v) polypeptone, 0.4% (w/v) K_2_HPO_4_, 3% (w/v) NaCl, and 0.5% (w/v) glycerol]. The gene under the control of the *araBAD* promoter was induced by addition of the appropriate amount of arabinose indicated. A solid and a soft agar medium were prepared by adding 1.25% and 0.25% agar, respectively. When necessary, the antibiotic chloramphenicol was used at 2.5 µg ml^−1^ for *V. alginolyticus* and 25 µg ml^−1^ for *E. coli* or the antibiotic ampicillin was added to a final concentration of 100 µg ml^−1^ for *E. coli*.

**Table 1:** Bacterial strains and plasmids used in this study.

Strain or plasmid	Genotype or description	Reference or source
*V. alginolyticus*		
VIO5	VIK4 (Rif^r^ Pof^+^ Laf^−^)	^36^
LPN1	VIO5 Δ*flhF* (Rif^r^ Pof^+^ Laf^−^)	^4^
*E. coli*		
DH5α	Recipient for DNA manipulation	
BL21(DE3)		Novagen
BL21(DE3)/pLysS		
Plasmids		
pBAD33	Cm^r^, P_BAD_	^37^
pTY57	Cm^r^, P_BAD_, MCS from pBAD24	^38^
pCold IV	Amp^r^, P_cspA_	Takara
pAK322	*flhF (wt)* in pBAD33	^3^
pAK325	*flhF-egfp (wt)* in pBAD33	^4^
pTSK110	*flhF-his6* in pCold IV	This study
pTSK119	*flhF-his6* in pTY57	This study
pSK102	*flhF (E440K)* in pBAD33	^21^
pSK111	*flhF (R334A)* in pBAD33	This study
pSK202	*flhF-egfp (E440K)* in pBAD33	^21^
pSK211	*flhF-egfp (R334A)* in pBAD33	This study
pSK301	*flhF-his6(G299A)* in pCold IV	This study
pSK302	*flhF-his6(G304A)* in pCold IV	This study
pSK303	*flhF-his6(K305A)* in pCold IV	This study
pSK304	*flhF-his6(T306A)* in pCold IV	This study
pSK305	*flhF-his6(K311A)* in pCold IV	This study
pSK306	*flhF-his6(D377A)* in pCold IV	This study
pSK307	*flhF-his6(G380A)* in pCold IV	This study
pSK308	*flhF-his6(T436M)* in pCold IV	This study
pSK309	*flhF-his6(K437A)* in pCold IV	This study
pSK310	*flhF-his6(D439A)* in pCold IV	This study
pSK311	*flhF-his6(E440K)* in pCold IV	This study
pSK312	*flhF-his6(D470A)* in pCold IV	This study
pSK313	*flhF-his6(R334A)* in pCold IV	This study
pTrc-flhG	*his6-tev-flhG* in pTrcHisB	^8^

Amp^r^, ampicillin-resistant; Cm^r^, chloramphenicol-resistant; Rif^r^, Rifampicin-resistant; Pof^+^, possessing a polar flagellum; Laf^−^, lack of lateral flagella.

#### Transformation of V. alginolyticus by electroporation

Overnight cultures were added to 40 ml of VC medium at 100-fold dilution and grown at 30 °C until OD 660 = 0.8. The cells were collected by centrifugation (3,000 × g for 5 min), suspended in 10 ml of Osmotic solution [30 mM Tris-HCl(pH8.0), 20% (w/v) sucrose, 400 mM NaCl, 1 mM EDTA] and grown with shaking at 30 °C for 10 minutes or more. After centrifugation (3,000 × g for 5 min), cells were suspended in 10 ml of 20 mM MgSO_4_ and allowed to stand on ice for 10 minutes. After centrifugation (3,000 × g for 5 min), cells were suspended in 200 to 400 µl of 10% (w/v) glycerol, and dispensed in 40 µl aliquots. Electroporation was carried out using a Gene Pulser electroporation apparatus (Bio-Rad Laboratories) with a 0.2 cm cuvette under the conditions of resistance 200 Ω and voltage 1.4 kV. After that, the bacteria were recovered in 1 ml of VC medium, grown with shaking at 30 °C for 40 minutes, then inoculated into VC agar plate containing chloramphenicol and grown overnight at 30 °C.

#### Purification of FlhF

For the expression of FlhF, a system using *E. coli* BL21 (DE3) strain was used. FlhF was expressed from a plasmid (pTSK110) in which *flhF* derived from *V. alginolyticus* was cloned into the NdeI-XhoI site of a cold shock expression vector pCold IV. The His-tag is designed to be added to the C-terminus of FlhF and the final product is expected to be a protein with 511 amino acids and a molecular weight of 57.4 kDa.

For small scale purfications, 5 ml of induced cells were centrifuged (16, 000 × g, 1 min) and washed with FlhF buffer [20 mM Tris-HCl(pH8.0), 300 mM NaCl, 10 mM MgCl_2_, 10 mM KCl]. The cells were suspended in 1.2 ml of FlhF buffer containing 5 mM GTP or GDP and the complete protease inhibitor, EDTA-free (Roche). The complete protease inhibitor was prepared by dissolving 1 tablet in 1 ml of sterilized water and used in a 50-fold dilution. Cells were sonicated using a small probe (power = 5, 30 sec, 3 times, duty cycle 50%) and undisrupted cells were precipitated by centrifugation at low speed (6,100 x g for 5 min). The supernatant was fractionated by ultracentrifugation (154,000 × g for 30 min) using a TLA55 rotor and Optima MAX (BECKMAN COULTER). One third of the soluble fraction was added to 100 µg of Ni-NTA resin (Qiagen) equilibrated with FlhF buffer containing 5 mM imidazole and mixed for 1 h at 4 °C using a rotator. After centrifugation to remove the solution, the 1/3 of sample was added to the resin. After mixing for 1 h, the third sample was also added to the resin in the same procedure. The resin was twice washed by 1 ml of FlhF buffer containing 5 mM imidazole. Then, 1 ml of FlhF buffer containing 30 mM imidazole was added to wash the resin three times. Thereafter, 300 μl of FlhF buffer containing 300 mM of imidazole and 10% (w/v) glycerol was added and incubated at room temperature for 5 min. After the centrifugation, the supernatant was recovered. The concentration of the purified protein obtained was calculated from a calibration curve prepared by SDS-PAGE with known concentration of BSA and quantification using the Coomassie Brilliant Blue (CBB) stained bands using Image J.

For large-scale preparation, 100 ml of culture was centrifuged (22, 200 × g for 5 min) and the collected cells were washed with FlhF buffer. The cells were suspended in 50 ml of FlhF buffer containing 0.5 mM GDP and one tablet of complete protease inhibitor (EDTA-free, Roche). Cells were sonicated using large probe (power = 5, 60 sec, 6 times, duty cycle 50%) and unbroken cells were removed by low speed centrifugation (6,300 × g for 5 min) The obtained supernatant was fractionated by ultracentrifugation (157,000 × g for 30 min) with a P50AT2 rotor using himac CP80WX (HITACHI). The soluble fraction was poured into a 5 ml disposable column (Thermo Scientific) packed with 1.5 ml of Ni-NTA resin equilibrated with FlhF buffer containing 5 mM imidazole. After all the soluble fraction passed through the column, the resin was twice washed by 5 ml of FlhF buffer containing 5 mM imidazole and washed by 5 ml of FlhF buffer containing 30 mM imidazole 3 times, then the proteins were eluted by 2.5 ml of FlhF buffer containing 300 mM imidazole and 10% (w/v) glycerol. The 200 μl aliquots of the eluted sample were frozen by liquid nitrogen and stored at −80 °C.

#### Measurement of GTPase activity

GTPase activity was measured using PiColorLock™ Gold Phosphate Detection System (Innova Bioscience). For analysis of FlhF alone, 40 μl of purified FlhF, 40 μl of FlhG buffer [20 mM Tris-HCl (pH8.0), 300 mM NaCl, 10% (w/v) glycerol] and 40 μl of GTP buffer [20 mM Tris-HCl (pH8.0), 300 mM NaCl, 10 mM MgCl_2_, 1.5 mM GTP, 10% (w/v) glycerol] were mixed and reacted for 30 min at 25 °C. For analysis of FlhF with FlhG, 40 μl of purified FlhF, 40 μl of FlhG and 40 μl of GTP buffer were mixed and reacted for 30 min at 25 °C. Final concentration of FlhF and FlhG were 1 μM. To stop the hydrolysis reaction, 30 μl of reaction terminator was added and after 2 min, 12 μl of the stabilizer was added. After standing at 25 °C for 30 min, the absorbance at 595 nm was measured by a plate reader (DS Pharma Biomedical), and the concentration of inorganic phosphate was calculated from a calibration curve obtained by similarly measuring the known inorganic phosphate. The measurements were performed for 3 purified samples by 3 measurements for one sample, thus the average value of n = 9 with standard deviation was shown as the activity measurement value.

#### Purification of FlhG

His-tagged FlhG was purified as described previously^8^. BL21 (DE3)/pLysS cells, transformed with pTrc-*flhG*, were cultured in 1 L of SB medium at 37 °C. At an OD 660 of 0.3 to 0.4, IPTG was added to a final concentration of 0.4 mM. After incubation at 37 °C for 3 hours, the cells were collected by centrifugation (6,600 × g for 10 min) and frozen at −80 °C. Frozen cells were suspended in 50 ml of FlhG buffer containing 5 mM imidazole and 0.5 mM PMSF and disrupted by sonication using a large probe (power = 8, 60 sec, 5 times, duty cycle 50%) and unbroken cells were removed by low speed centrifugation (6,300 × g for 5 min). The supernatant was fractionated by ultracentrifugation (157,000 × g for 30 min) with the P50AT2 rotor using himac CP80WX (HITACHI). The soluble fraction was passed through a HiTrap TALON column (5 ml) connected to the AKTA PRIME system. After washing the column by FlhG buffer containing 20 mM imidazole at a flow rate of 1.0 ml/min, FlhG was eluted with FlhG buffer containing 65 mM imidazole at a flow rate of 1.0 ml min^−1^. The 100 μl aliquots of the eluted sample were frozen by liquid nitrogen and stored at −80 °C.

#### Detection of FlhF protein

The samples were mixed with one-fifth volume of SDS loading buffer [0.2 M Tris-HCl (pH 6.8), 37.5% (w/v) glycerol, 6% (w/v) SDS, and 0.004% (w/v) bromophenol blue] and one-tenth volume of 2-mercaptoethanol, and then boiled for 5 min. Proteins in the samples were separated by SDS-PAGE, and immunoblotting was conducted as described previously^21^, using the anti-FlhF antibody.

### Sequencing

Reactions for DNA sequencing were performed by a dye terminator method using BigDye Terminator v3.1 Sequencing Standard Kit. The Nagoya University DNA sequencing core facility performed the DNA sequence analysis. Data were analyzed using GENETYX and 4 peaks software.

### Site-specific mutagenesis using PCR

Routine DNA manipulations were carried out according to standard procedures. The mutations of *flhF* were introduced into plamids by the QuikChange site-directed mutagenesis method using a pair of primers for the desired mutation (Table S3). To amplify DNA, Pfu ultra polymerase was used, and the reaction cycle was 95 °C for 30 sec → 18 cycles of (95 °C for 1 min → 55 °C for 1 min → 68 °C for16 min) → 4 °C. Dpn I treatment was carried out to eliminate the template plasmid and introduced into *E. coli* DH5α. The plasmid was isolated from transformed cells and the nucleotide sequence of *flhF* was determined to confirm the mutation.

### Detection of flagella in *Vibrio* cells

Overnight cultures were diluted 1:100 in VPG medium containing 0.02% (w/v) arabinose and 2.5 μg ml^−1^ chloramphenicol and further cultivated at 30 °C for 4 hours. The cultured cells were harvested and suspended in V buffer [50 mM Tris-HCl (pH7.5), 300 mM NaCl, 5 mM MgCl_2_]. Flagella were observed under a high-intensity dark-field microscope (Olympus model BHT) equipped with a 100-W mercury lamp (Ushio USH-102).

### Observation of GFP fluoresence in cells

*Vibrio* cells were cultured overnight in VC medium at 30 °C. Overnight cultures were diluted 1:100 in a fresh VPG medium containing 0.02% (w/v) arabinose and 2.5 μg ml^−1^ chloramphenicol and cultivated further at 30 °C for 4 hours. The cells were collected by centrifugation (97,700 × g for 2 min), and suspended to V buffer at 1/4 volume of original culture. Fluorescence microscopic observations were carried out as described previously^8^.

### Estimation of particle size by gel filtration chromatography

GTP or GDP was added to the purified FlhF protein to a final concentration of 1 mM, and the mixture was allowed to stand on ice for 10 minutes. A 100 μl portion of the protein solution was loaded on a SuperDex 75 Increase column (GE Healthcare) that had been equilibrated with buffer [20 mM Tris-HCl (pH 8.0), 300 mM NaCl, 10 mM MgCl_2_] containing 1 mM GTP or GDP, and eluted by the same buffer at a flow rate of 0.5 ml min^−1^. The elution was collected in 0.5 ml fractions and the elution position of the protein was confirmed by western blot.

## Electronic supplementary material

Supplemental information
