# Supplementary material for: Biochemical analysis of GTPase FlhF which controls the number and position of flagellar formation in marine Vibrio
Source: Sci Rep. 2018 Aug 14;8:12115. doi: 10.1038/s41598-018-30531-5 (PMC6092412; doi:10.1038/s41598-018-30531-5)
Supplement: Supplementary file 1 — Supplemental information [file 41598_2018_30531_MOESM1_ESM.pdf]

**Supplementary information**

**Biochemical analysis of GTPase FlhF which controls the number and position of flagellar formation in marine *Vibrio*.**

Shota Kondo, Yoshino Imura, Akira Mizuno, Michio Homma*, and Seiji Kojima

Division of Biological Science, Graduate School of Science, Nagoya University, Chikusa-ku, Nagoya 464-8602, Japan

*Corresponding author

# Fig. S1. Kondo *et al.*

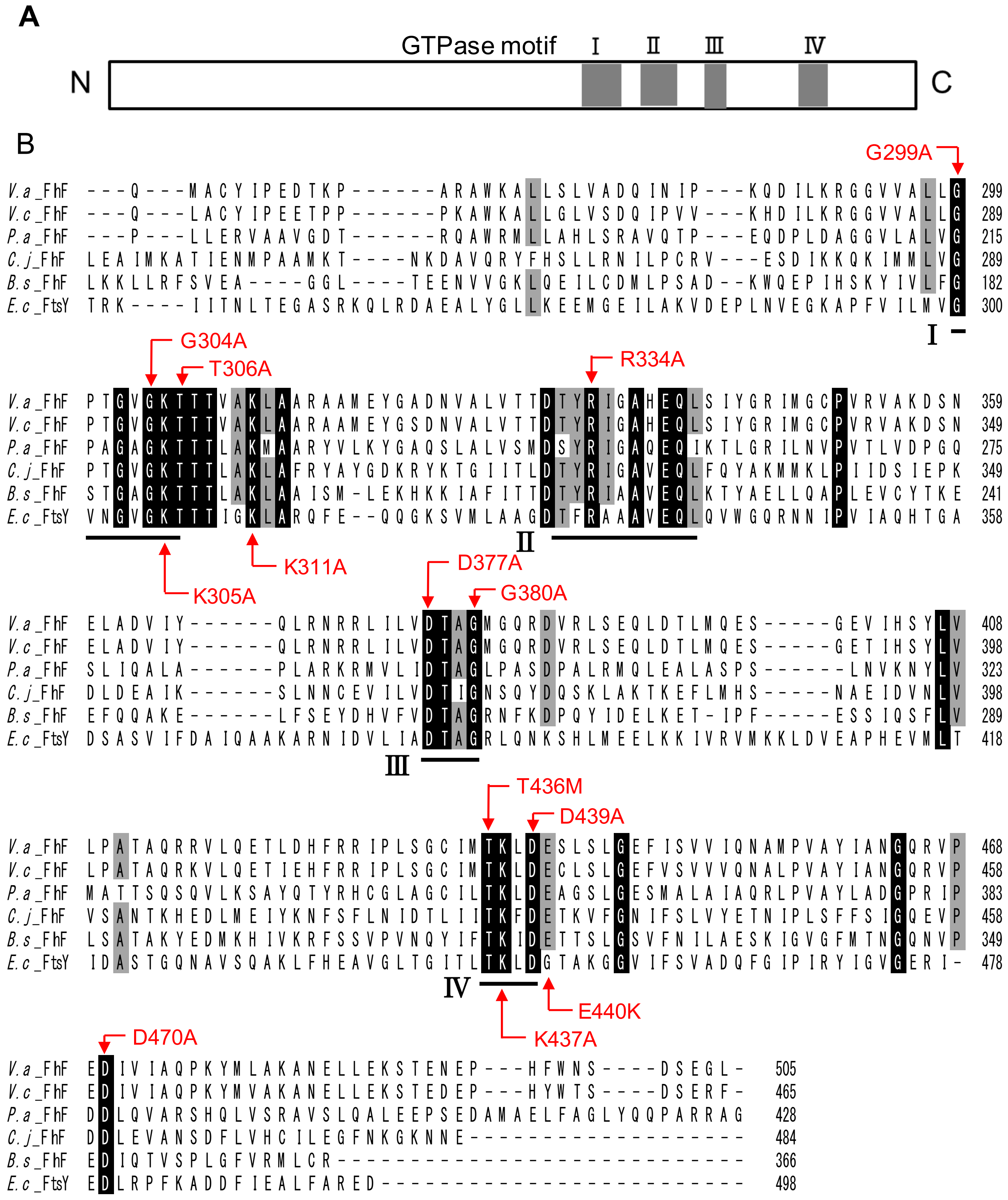


**Fig. S1.** (A) Schematic diagram of the primary structure of *Vibrio* FlhF with GTPase motif. (B) Alignments of the C-terminal regions where the GTPase motifs are present. The mutant residues used in this study are indicated by red arrows and the GTPase motifs are underlined. The amino acid sequences are derived from V.a: *Vibrio alginolyticus*, V.c: *Vibrio cholerae*, P.a: *Pseudomonas aeruginosa*, C.j: *Campylobacter jejuni*, B.s: *Bacillus subtilis*, E.c: *Escherichia coli*.

# Fig. S2. Kondo *et al.*

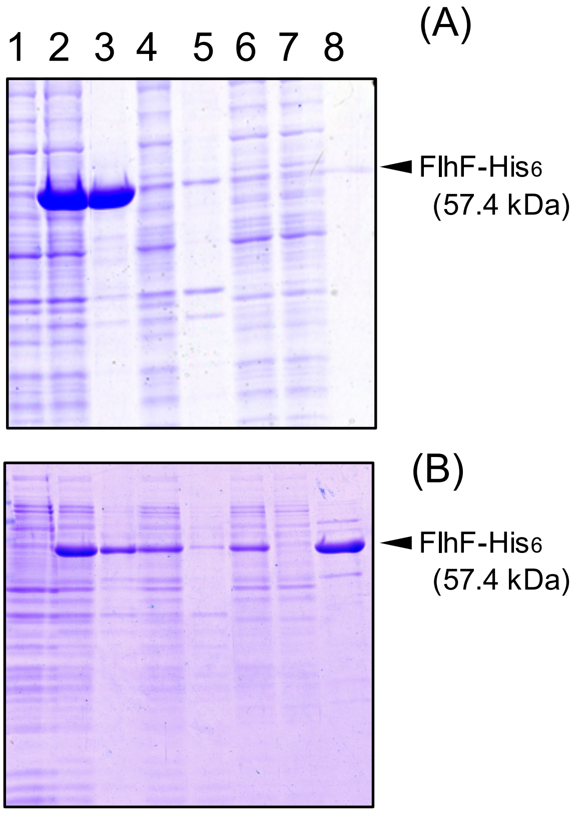


**Fig. S2.** Cells of BL21(DE3) harboring a plasmid pTSK110 which has *flhF-his6* in pColdIV vector were broken in the presence (B) and absence (A) of $MgCl_2$ and GTP. 1: the pre induction cells, 2: the post induction cells, 3: the low speed centrifugal precipitation, 4: the low speed supernatant, 5: the ultracentrifugation precipitation, 6: the ultracentrifugal supernatant, 7: the flow through fraction of Ni-NTA resin, 8: the eluted fraction of Ni-NTA resin. The proteins were separated by SDS-PAGE and the gels were stained by CBB.

Fig. S3. Kondo *et al.*

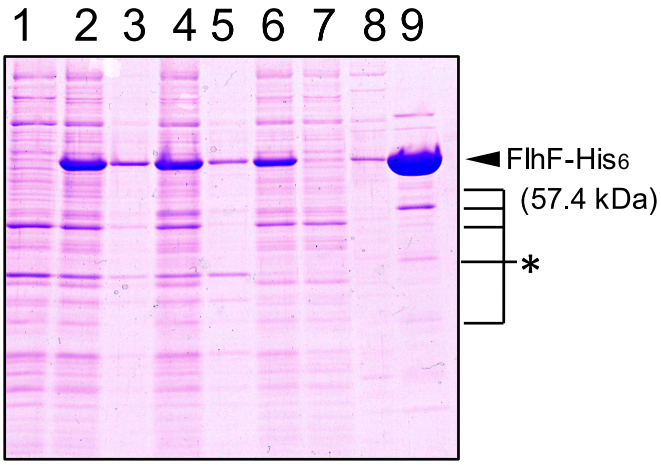


**Fig. S3.** Cells of BL21(DE3) harboring a plasmid pTSK110 which has *flhF-his6* in pColdIV vector were broken in the presence of $MgCl_2$ and GDP for large scale preparation established in this study. The gel after SDS-PAGE was stained with CBB. * is degradation products of FliF-His6. 1: the pre induction cells, 2: the post induction cells, 3: the low speed centrifugal precipitation, 4: the low speed supernatant, 5: the ultracentrifugation precipitation, 6: the ultracentrifugal supernatant, 7: the flow through fraction of Ni-NTA resin, 8: the washed fraction of Ni-NTA resin, 9: the eluted fraction of Ni-NTA resin.
